# The effect of autistic traits on response to and side-effects of pharmacological ADHD treatment in children with ADHD: results from a prospective clinical cohort

**DOI:** 10.1186/s11689-022-09424-2

**Published:** 2022-03-06

**Authors:** Maria M. Lilja, Emil Sandblom, Paul Lichtenstein, Eva Serlachius, Clara Hellner, Jyoti Bhagia, Linda Halldner

**Affiliations:** 1 Department of Clinical Sciences, Child and Adolescent Psychiatry, Umea University, Universitetstorget 4, SE907 36 Umea, Sweden; 2 Child and Adolescent Psychiatric Clinic, Stockholm, Sweden; 3 Department of Medical Epidemiology and Biostatistics, Karolinska Institutet, PO Box 281, SE-171 77 Stockholm, Sweden; 4 Department of Clinical Neuroscience, Center for Psychiatry Research, Karolinska Institutet, PO Box 281, SE171 77 Stockholm, Sweden; 5 Stockholm Health Care Services, Region Stockholm, Sweden; 6 Department of Psychiatry and Psychology, Mayo Clinic, 200 First Street SW, Rochester, MN 55905 USA

**Keywords:** ADHD, ASD, Pharmacological treatment, Effect, Adverse event

## Abstract

**Background:**

Attention deficit hyperactivity disorder (ADHD) is a common childhood behavioral condition that globally affects an average of around 5% of children and is associated with several adverse life outcomes. Comorbidity with autism spectrum disorder (ASD) is highly prevalent. Pharmacological treatment for ADHD symptoms has been shown to be effective. However, the prevailing perception is that children with ADHD and concomitant ASD symptoms report poorer efficacy and more side effects. This has been supported by studies on this population, but prospective studies directly comparing children with ADHD and different levels of ASD symptoms are lacking. We aimed to assess if children with ADHD and concomitant ASD symptoms differ regarding effects and side-effects of pharmacological ADHD treatment compared to children with ADHD without ASD traits. This is to our knowledge the second study to directly compare the effect of ADHD medication between ADHD patients with different levels of ASD symptoms.

**Methods:**

In a non-randomized, observational, prospective cohort study, 323 patients aged 6 to 17 years who were diagnosed with ADHD and starting pharmacological treatment were divided into two groups: one with high level of ASD symptoms (ASD group, *N*=71) and one with low level of ASD symptoms (non-ASD group, *N* = 252). Treatment outcome was measured as ADHD symptoms, and evaluated using the Swanson, Nolan and Pelham Teacher and Parent ADHD rating scale-version IV (SNAP-IV). Side-effects were evaluated using the Pediatric Side Effects Checklist (P-SEC), at 3 months follow-up.

**Results:**

From baseline to 3 months, there was no significant difference in neither treatment effect nor number of clinically significant adverse events experienced between the ASD group and the non-ASD group.

**Conclusions:**

Our results did not implicate that ADHD patients with concomitant ASD symptoms have decreased treatment effect of ADHD medication than patients with ADHD without concomitant ASD symptoms. Neither did the results support that ADHD patients with ASD symptoms experienced significantly more side-effects than ADHD patients without ASD symptoms. Although, we did not analyze different medications separately, this is in line with the only previous study directly comparing methylphenidate treatment in children with or without ASD.

**Trial registration:**

NCT02136147, May 12, 2014.

## Background

Attention deficit hyperactivity disorder (ADHD) is a common childhood behavioral condition that globally affects an average of around 5% of children [1]. There is a high degree of co-morbidity between ASD and ADHD [2]. Studies show between 5 and 80% of ADHD patients also show features of ASD or have comorbid ASD diagnosis. The reverse relation, ASD with elevated levels of ADHD symptoms or ADHD diagnosis varies between 30 and 75% [2–6]. For ADHD, pharmacological treatment is the primary therapeutic strategy. Stimulants, e.g., methylphenidate (MPH) or dexamphetamine (DEX), usually are the first drugs of choice. The effect of different pharmacological substances in children with ADHD has been widely examined [7–10], which suggests that medication has pronounced effect on typical ADHD manifestations and decreases the risk of detrimental life events [11]. However, a common view in clinical practice that is also supported in earlier studies is that children with co-morbidity between ASD and ADHD often respond poorly to standard ADHD treatment and/or have increased side effects [12–14], which results in higher discontinuation rates [15, 16]. The assumption of decreased treatment effect and increased side-effects of ADHD medication in children with ASD derives from comparisons of results from studies on children with only ADHD, with results from studies on children with ASD and comorbid ADHD [2, 17]. Regarding response rate, a 2005 randomized crossover trial of MPH in pervasive developmental disorders (PDD) with hyperactivity showed a 50% response rate in individuals with ASD and ADHD [15], while in 1999 Jensen et al. presented a 70–80% response rate in individuals with only ADHD [16]. In spite of the lower response rate to MPH in individuals with ASD and ADHD as compared to individuals with ADHD without ASD, MPH is recommended for ADHD symptoms in children with ASD [18]. Further, regarding stimulants, which are the most studied ADHD medications, one meta-analysis from 2010 found MPH to be effective in children with ADHD [19], whereas another meta-analysis from 2013 found MPH to be effective in PDD patients with ADHD [20]. However, in 2016, Accordino et al. presented heterogenous results of stimulants in an ASD population [12]. Varying effect sizes makes comparison difficult. Regarding non-stimulant ADHD medications, a systematic review showed atomoxetine (ATX) to be effective in ADHD patients [21], even though Newcorn et al. displayed a more subtle effect [22, 23], ATX studies also indicate improvement in ADHD symptoms, at least hyperactivity, in children with comorbid ASD and ADHD [24–27]. Two RCT studies by Scahill et al. demonstrated guanfacine (GXR) to be effective in children with ADHD and ASD [28, 29].

Reichow et al. performed a systematic review and meta-analysis of 7 double-blind randomized placebo-control trials in which they examined the effect of ADHD medication in children with PDD [20]. The authors concluded common side-effects, such as insomnia and decreased appetite, occurred at similar rates in PDD population, whereas side effects of depression, irritability, and social withdrawal appeared to be more common in children with PDD compared to studies on children with ADHD without PDD. Accordino et al. [12], investigating existing evidence for medications for ADHD-type symptoms in children with ASD, found stimulants to be an option but also potentially problematic given the vulnerability to increased irritability. Accordino et al. refers to the RUPP study (children with PDD and hyperactivity) [15] and Greenhill et al. (children with ADHD without ASD) [30], where side-effects were more common in the RUPP study, with a discontinuation rate of 18%, mostly due to irritability. In children with ADHD without ASD, Jensen et al. reported considerably lower discontinuation rates: 1.4% [16]. On the contrary, a recent study from Ventura et al., did not find a significant difference in discontinuation rates between ADHD patients and ADHD + ASD patients [31]. However, adherence rate was preserved by strictly monitored follow-up. A Cochrane review of the effects of MPH (including 5 crossover studies of children with ADHD and comorbid ASD) concluded that the evidence for adverse events had poor quality, since trials were short and with small sample size [32].

Thus, even though studies examining the pharmacological treatment effect of various ADHD medications in children with ASD exist, there is a shortage of clinical trials conducted on children with or without concomitant ASD symptoms comparing treatment effect and side effects. We have identified only one study directly comparing children with ADHD without ASD diagnosis to children with ADHD and concomitant ASD [33]. Santosh et al. initially performed a retrospective chart review comparing the results of ADHD medication in 113 children with pure ADHD and 61 children with concomitant ASD and ADHD. Finding no significant differences in effect and side-effects, they continued with a prospective observational study design comparing ADHD treatment results from children with (*n* = 27) and without (*n* = 25) concomitant ASD diagnosis. No statistically significant differences between the groups were found [33]. In the present study, we aimed to study any differences in treatment outcome in a larger clinical prospective study cohort of children with or without high levels of ASD symptoms.

## Methods

### Study design and research participants

Research participants in this study constitute a subsample of those enrolled in the ADHD medication and predictors of treatment outcome (ADAPT) study, a prospective observational cohort study conducted in three Swedish Child and Adolescent Psychiatry units: in Stockholm, Gotland, and Umeå. The ADAPT study has enrolled patients from July 2014 and is still running. By January 2020, 548 patients were included. Patient enrolment in Umeå started in March 2020, and thus for the present all study participants were from the Swedish Child and Adolescent Psychiatry units in Stockholm and Gotland. Inclusion criteria for the ADAPT study were (1) age 6 to 17 years, (2) established clinical ADHD diagnosis, and (3) initiating pharmacological ADHD treatment. Patients who had been on any ADHD medication within the last 3 months prior to study start were not eligible. Participating units were instructed to ask all eligible patients on participation and to report numbers declining and accepting to the study. However, only the numbers of accepting participants were reported. For the present study, participants lacking baseline rating of ASD-symptoms, baseline rating of ADHD-symptoms, or rating of ADHD-symptoms at 3 months were excluded. Forty-three patients did not complete the Autism Spectrum Screening Questionnaire (ASSQ) at baseline, 40 patients did not complete SNAP-IV at baseline, and 208 patients did not complete SNAP-IV at 3 months, leaving the cohort with 323 patients. 295 patients in the cohort had complete information on ADHD medication at 3 months; information was missing for 28 participants. One 5-year old was accepted in the cohort.

**Fig. 1:**
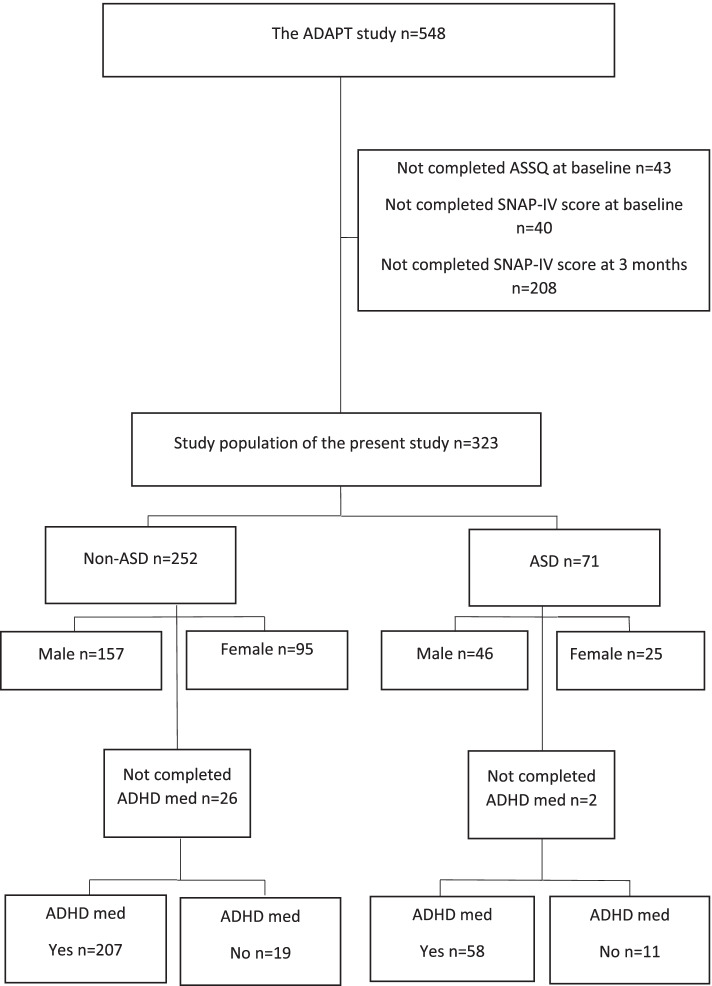
Participant Flowchart

Participant flowchart is presented in Fig. 1. See Table 1 for demographic characteristics at baseline and ADHD medication at 3 months.

**Table 1:** Demographic and clinical characteristics of the study participants

Child characteristics	All research participants (***N*** = 323)		Diff. between groups (***P*** value)
Baseline measures			
	Non- ASD symptoms (***n*** = 252)	ASD symptoms (***n*** = 71)	
Age, year, mean^a^	11.75	10.26	.001
Age range, year^a^	5–17	6–17	
Gender, male, *n* (%)^a^	157 (62.30)	46 (64.79)	.65
Weight, kg, mean, (95% CI)^a^	48.05 (45.77–50.34)	43.97 (39.59–48.36)	.110
Length, cm, mean, (95% CI)^a^	152.7 (150.55–155.01)	145.10 (141.96–150.03)	.003
SNAP-IV, mean, total score, (*n*)^b^	43.07	57.06	<.001
ASSQ, mean, total score (*n*)^b^	7.46	22.71	<.001
ASSQ, median (*n*)^b^	7	20.62^f^	
**ADHD medication at 3 months (%)**^**c**^			
No ADHD medication (*n* = 30)^c^	8.39 (19)	16.03 (11)	
ADHD medication (*n* = 265)^c^	91.61 (207.4)	83.97 (57.6)	
Pearson chi-square (*P*)	.06623		
**ADHD medication at 3 months Subdivided in substances**^**d**^**(%)**			Pearson Chi-Square (*P*)^e^
MPH	74.95 (153.2)	77.39 (43.8)	.68
DEX	0	0	
LDX	15.19 (31.2)	15.55 (8.8)	.84
ATX	9.25 (19)	5.30 (3)	.36
GXR	.49 (1)	3.53 (2)	.05

^a^Unpooled data after ASSQ imputation

^b^Pooled data after multiple imputation

^c^Pooled data after multiple imputation of ASSQ and SNAP IV scores at baseline and at 3 months. (Non-ASD symptoms *N* = 226.4 and ASD symptoms *N* = 68.6)

^d^*MPH* Methylphenidate, *DEX* Dexamphetamine, *LDX* Lisdexamphetamine, *ATX* Atomoxetine, *GXR* Guanfacine

^e^Chi-square test is calculated as a mean of chi-square for original data and imputation 1–5, since it is not possible to get chi-square for pooled values after multiple imputation

^f^Mean for original data and 5 imputations

### Measures

To estimate the effect of medication and assess the ADHD symptoms, we used parent’s ratings of the validated Swanson, Nolan and Pelham Teacher and Parent ADHD rating scale-version IV (SNAP-IV) [34, 35] at baseline and at 3 months follow-up. The SNAP-IV consists of a 4-point Likert scale where parents rated their child’s various symptoms as: 0 (not at all), 1 (just a little), 2 (quite a bit), or 3 (very much). Thirty items constitute the scale, of which twenty-six can be divided into three subscales: inattention (items 1–9), hyperactivity/impulsivity (items 11–19), and oppositional defiant disorder (ODD) (items 21–28). The remaining questions are of more summarizing character. The total range of score is 0 to 90.

To answer our main research question, we compared the total SNAP-IV points at baseline and at 3 months follow-up, although we also performed analyses on the subscales. Further, we introduced a prerequisite, a cutoff at 40% reduction in total SNAP-IV score from follow up at 3 months compared to baseline. This resulted in two groups: responders, defined as a SNAP-IV score reduction ≥ 40 %, and non-responders, defined as a SNAP-IV score reduction < 40%. According to previous studies, a 40% threshold has shown good coherence with the degree of change in the SNAP-IV rating scale score that predominantly correspond to a substantial clinical effect in regards of ADHD characteristic symptoms [22, 23].

To distinguish children in our cohort with high level of ASD symptoms, we used the validated Autism Spectrum Screening Questionnaire (ASSQ) [36, 37]. The ASSQ comprises twenty-seven items regarding assertions of core ASD symptoms. Parents rate their child on a 3-point Likert scale: 0 (no), 1 (somewhat), or 2 (yes). The range of scores is 0 to 54. Eleven items tap topics regarding social interaction, six cover communication problems, five refer to restricted and repetitive behavior, and five embrace motor clumsiness and tics. ASSQ scores were registered at baseline to identify children with more pronounced ASD symptoms from those with less pronounced symptoms. Cut-off was set to a total of 17 points, where children who scored 17 or above were regarded as the ASD group [38]; consequently, children with scores below 17 were assessed as the non-ASD group. The creators of the questionnaire originally recommended 19 as the cut-off [37] when used in a psychiatric setting, although later studies have shown 17 to be a good tradeoff between sensitivity and specificity [39].

The Pediatric Side Effects Checklist (P-SEC) [40], contains 50 items split into 11 categories reflecting adverse effects of medicine, encompassing the gastrointestinal system, the central nervous system, the endocrine system, mood/behavioral changes, the cardiovascular system, the immune system, the skin, the renal system, sexual concerns/problems, allergic reactions, and other symptoms. The items in the other symptoms category could be further specified in free text. For our analysis, we included only the 49 items with numeric values. For each of the 49 items, the parent rated their child’s manifestations on a 4-point scale: 0 (none), 1 (mild/sometimes but tolerable), 2 (moderate/interferes somewhat), or 3 (severe/interferes a lot). The maximum score on the scale is 147. P-SEC was accomplished at baseline and at 3 months follow-up. To assess the number of clinically adverse events present at 3 months after start of pharmacological ADHD treatment, we grouped all 49 items in the P-SEC scale into the 11 subcategories in accordance to affected organ system. Adverse events were noted as a binary categorical variable, present (1 = yes) or absent (0 = no). In order to estimate only clinically significant side-effects, an item was recognized as an adverse event only if the score at follow-up was 2 or more, (the minimum for not tolerable side-effects and thus probably comparable to earlier studies suggestions on higher level of side-effects leading to treatment discontinuation), provided that the score had increased since baseline registration. A similar rationale to recognize only significant worsening was acknowledged by Simonoff et al. [41], (using a 2 point cut-off deterioration from baseline [42]).

Initiating ADHD medication was an inclusion criterion for enrollment in the study. Consequently, all patients were assumed to have started ADHD medication at baseline. At 3 months, data on ADHD medication was obtained and noted as binary yes or no answers. ADHD medication was defined as at least one of either methylphenidate (MPH), dexamphetamine (DEX), lisdexamphetamine (LDX), atomoxetine (ATX), or guanfacine (GXR). In Sweden, MPH is the first drug of choice and accordingly was prescribed to the vast majority of patients. Thus, hereafter when we refer to ADHD medication, it indicates the use of MPH, LDX, ATX, or GXR alone or in combination with others, though the medication used was predominantly MPH. The distribution of ADHD medication (MPH, LDX, ATX, and GXR) is presented in Table 1. None of the patients in our cohort were treated with DEX at 3 months follow-up. Our aim for this study was not to compare the effect of different ADHD medications, and analyses for separate substances were not performed.

### Data analysis

To examine whether patients in the ASD group had inferior treatment effect and augmented adverse effects of ADHD medication compared to patients in the non-ASD group, we analyzed the differences between the ASD group and the non-ASD group concerning total SNAP-IV score reduction from baseline to 3 months as well as a number of side effects for each group.

A multivariable linear regression model was conducted to assess the relationship between the dependent outcome variable, SNAP-IV score at 3 months, and the independent variables (ASD group vs. non-ASD group), adjusting for age, gender, and baseline SNAP-IV score.

In addition, we also performed logistic regressions to assess if the interaction between variables would change if we set the primary outcome to a binary variable, responder or non-responder, using the same independent variables: ADHD medication, ASD or non-ASD group, age, gender, and SNAP-IV score at baseline.

The sum of the number of adverse events in each of the 11 categories from the P-SEC scale was calculated and compared between the non-ASD group and the ASD group using chi-square tests with continuity correction.

All statistical analyses were conducted using IBS SPSS Statistics software version 26. Comparisons were 2-tailed with statistical significance set at *P*-.05.

### Missing data

Multiple imputations (Mis) technique was used to limit the effect of occasional single missing data in ASSQ and SNAP-IV questionnaires [43, 44]. Mis was conducted using IBS SPSS Statistics 26. Mis uses patterns in the available data to make a probability judgement to what the missing value most likely would be and replaces it. Missing values were randomly distributed and did not exceed 5% of the whole data. All SNAP-IV scales, both at baseline and at 3 months, and ASSQ scale variables were included in the Mis model (a total of *n* = 87 datapoints). The number of random lacking data points were distributed according to SNAP-IV at baseline (*n* = 31), SNAP-IV at 3 months (*n* = 43), and ASSQ at baseline (*n* = 36). Mis generates 5 imputations and thereof a merged pooled result. All analyses were conducted following imputation.

Mis generates a pooled non-integer value, which therefore is not compatible with all analyses in IBS SPSS Statistics software. Since the non-ASD and ASD group were defined by a pooled multiple imputed value of the ASSQ data, the groups were not absolute determined entities. Using pooled data, the non-ASD group was actually *n* = 252.4 and the ASD group was *n* = 70.6. This fact had a negligible effect on our results but did result in somewhat varying composition of the non-ASD and ASD group for dichotomous analyses, precisely examining adverse events, and SNAP-IV responder and non-responder. However, importantly, it did not have any significant impact on our findings.

Concerning the P-SEC scale, random missing data counts were regarded as 0, since we assumed leaving out answering solitary questions about adverse events almost exclusively could be viewed as having “no side-effect,” which equaled a rating of 0. Multiple imputation method used for the SNAP-IV scale and the ASSQ scale was not considered applicable, since the items on the P-SEC scale represent very different themes, and consequently the imputation model would adventure generating adequate answers. There were on average 2.62 (95% CI 1.57–3.68) missing data points per patient in the P-SEC ratings in the Non-ASD group and 2.16 (95% CI 0.69–3.63) per patient in the ASD group. Consequently, the low number of missing data points on the P-SEC scale did not motivate the risk of bias trying to adjust for missing data.

## Results

Independent *t* test established that the non-ASD group scored significantly lower on the SNAP-IV scale than the ASD symptoms group. When comparing total SNAP-IV score, at baseline the mean difference between groups was 13.99 points (CI: 9.39–18.59; *P* < .001), and at 3 months, the mean difference was 8.72 points (CI: 4.32–13.11; *P* < .001). Both groups, as expected, decreased in scores from baseline to 3 months. Accordingly, the same pharmacological treatment effect between the two groups remained when adjusted for higher scores for the ASD symptoms group. When analyzing each SNAP-IV subscales separately—inattention, hyperactivity/impulsivity, and ODD—the same pattern with lower scores for the non-ASD group compared to the ASD group, including both at baseline and at 3 months, as well as the sum of scores overall deceasing from baseline to 3 months for both groups, were exhibited. A slight difference between subscales was observed, where the change in inattention scores (mean difference at baseline 2.38 (*P* = 0.01) and 1.84 (*P* = .015) at 3 months) was less pronounced between the groups than for hyperactivity/impulsivity (mean difference at baseline 5.34 (*P* < .001) and 2.79 (*P* = .001) at 3 months) and ODD (mean difference at baseline 4.31 (*P* < .001) and 2.81 (*P* < .001) at 3 months). The divergence between the non-ASD and the ASD group was significant throughout all subscales except for inattention score at 3 months (Table 2).

**Table 2:** SNAP-IV scores for each subscale; inattention, hyperactivity/impulsivity, and oppositional defiant Disorder (ODD)

SNAP-IV	Non-ASD symptoms (***n*** = 252)	ASD symptoms (***n*** = 71)	Mean difference (***n***) 95% Confidence interval	***t*** test significance (***p***)^**1**^
Total score, baseline, mean (*n*)	43.07	57.06	13.99 (CI;9.39-18.59)	< .001*
Total score, 3 months, mean (*n*)	31.13	39.85	8.72 (CI; 4.32–13.11)	< .001*
Inattention score, baseline, mean (*n*)	17.73	20.11	2.38 (CI; 0.97–3.79)	.001*
Inattention score, 3 months, mean (*n*)	12.57	14.42	1.84 (CI; 0.35–3.34)	.015
Hyperactivity/impulsivity score, Baseline, mean (*n*)	12.40	17.73	5.34 (CI; 3.33–7.35)	< .001*
Hyperactivity/impulsivity score, 3 months, mean (*n*)	8.73	11.51	2.79 (CI; 1.07–4.59)	.001*
ODD score, baseline, mean (*n*)	9.55	13.86	4.31 (CI; 2.61–6.01)	< .001*
ODD score, 3 months, mean (*n*)	7.35	10.19	2.81 (CI;1.29–4.39)	< .001*

Table displaying SNAP-IV scores, at baseline and at 3 months respectively, distributed between the non-ASD symptoms group and the ASD symptoms group

^1^Bonferroni correction was applied for the multiple t-tests. Post-hoc Bonferroni correction α = .003. Thus, adjusted significance level for *p*-value was < .003

*Significant *p* value

At 3 months, 265 patients declared having ADHD medication and 30 patients stated not. In the non-ASD group, 8.39% of participants (*n* = 19) registered as having no ADHD medication and 91.61% (*n* = 207.4) registered as having ADHD medication at 3 months. In the ASD group, 16.03% of participants (*n* = 11) were noted as having no ADHD medication, and 83.97% (*n* = 52) did have ADHD medication at 3 months (*P* = .06623). 74.95% (*n* = 153.2) in the Non-ASD group and 77.39% (*n* = 43.8) in the ASD group stated treatment with MPH (*P* = .68). The second most frequent treatment in both groups was LDX, 15.19% (*n* = 31.2) in the non-ASD group and 15.55% (*n* = 8.8) in the ASD group (*P* = .84) (Table 1).

SNAP-IV at baseline predicted SNAP-IV at 3 months (beta = 0.545, 95% CI: 0.451 to 0.638, *P* < .001). The effect of ADHD medication on SNAP-IV score at 3 months indicated that the medicated group had a lower score, albeit not significant (− 5.130, 95% CI: − 10.298 to .038, *P* = 0.052), after controlling for age and gender (Table 3). Thus, in our model, there was no significant difference in total SNAP-IV score outcome at 3 months between the ASD group and the non-ASD group. Similarly, there was no compelling evidence indicating discrepancy in efficacy of ADHD medication between the non-ASD and the ASD group when examining the dichotomous variable, responder, and non-responder. In the non-ASD group, 35% (*N* = 88.8) were responders and 65% (*N* = 163.6) were non-responders. In the ASD-group, 30% (*N* = 21) were responders and 70% (*N* = 49.6) were non-responders. Pearson chi-square test (median of original data and 5 imputations) with continuity correction did not reveal a significant difference (*P* = .447). The logistic regression analyses did not show any significant interactions between any of the variables. Chi-square tests with continuity correction, performed to examine the relation between ASD symptoms and adverse events, did not exhibit significant difference of reported adverse events for any of the 11 subcategories between the non-ASD and the ASD group (Table 4).

**Table 3:** Linear regression analysis

Coefficients						
Explanatory variable	Unit of measurement	Unstandardized coefficinets			95% confidence interval forB	
		B	Std. error	Sjg.	Lower bound	Upper bound
Constant		13.565	5.463	.013	2.858	24.273
Total SNAP-IV score at baseline	Number	.545	.048	.000*	.451	.638
Non-ASD/ASD	Categorical (1 = non-ASD, 2 = ASD)	.695	1.993	.727	−3.211	4.601
ADHD medication	Categorical (0 = no, 1 = yes)	−5.130	2.637	.052	−10.298	.038
Gender	Categorical (1 = male, 2 = female)	1.638	1.725	0.342	−1.743	5.019
Age (year)		−.386	.271	.154	−.917	.145
	Number					

Table displaying the relationship between the dependent outcome variable, SNAP IV score at 3 months, and ASD symptoms/Non-ASD symptoms, as independent variable. Adjusting for age, gender, and baseline SNAP-IV score

*Significant at .01; *R*^2^ = .389

**Table 4:** Adverse events

Subcategory Organ system^b^	Study Group				Chi-square test with continuity correction (***P***)
	Non-ASD (***N*** = 257)^**a**^		ASD (***N*** = 66)^**a**^		
	No significant symptoms (n)	Significant symptoms (n)	No significant symptoms (n)	Significant symptoms (n)	
Gastrointestinal	138	119	40	26	.385
Central nervous system	130	127	33	33	1.0
Endocrine	196	61	48	18	.663
Mood/behavior changes	225	32	52	14	.105
Cardiovascular	247	10	61	5	.347*
Immune system	253	4	65	1	1.0*
Skin	243	14	63	3	1.0*
Renal	250	7	64	2	1.0*
Sexual	256	1	66	0	1.0*
Other	252	5	64	2	.947*
Allergic	219	38	55	11	.851

Table displaying number of reported significant symptoms and No significant symptoms, for the Non-ASD symptoms group and the ASD symptoms group, subdivided in 11 organ systems

*The chi-squared approximation may be incorrect due to insufficient number

^a^The different number of individuals in the groups are due to multiple imputation. Using pooled values defining the groups, results in non-integer numbers. The chi-square tests are analyzed from pooled values

^b^The total range of score on the P-SEC scale is 0 to 196. However, in our analyses, the range was 0–49, after changing the items into dichotomous variables

## Discussion

Our results indicate that there were no differences in pharmacological ADHD treatment effect between patients with ADHD and concomitant high level of ASD symptoms and patients with ADHD and low level of ASD symptoms. In addition, the results from this study indicate that patients with ADHD and high level of ASD symptoms did not experience more adverse events from medication as compared to patients with ADHD and low level of ASD symptoms.

In contrast to our results, previous studies have concluded that children with ASD have less effect of pharmacological treatment of ADHD symptoms and experience more adverse events [15, 20]. These conclusions were, however, drawn comparing results from studies on children with ASD to studies performed on children without ASD, rather than, as is the case for this study, directly comparing the groups themselves. For instance, increased side-effects were not reported or not significantly increased in studies with uncomplicated ADHD. Indeed, overall, it was difficult to compare studies due to the use of disparate rating-scales and outcome measures. For example, the response rate in the present study was generally low, which may be due to that the definition for response was set fairly high. Previous studies that have used this definition of treatment response [22, 23], also reported lower response rates than often reported from RCTs [10]. Also, this clinical observational study had few exclusion criteria and thus may represent a more heterogenous cohort than in most RCTs. This may also affect the observed response rate. An international consensus concerning standardized outcome scales or measures would facilitate comparisons in future studies.

It is also possible that the low response rates observed in the present study are due to non-optimal pharmacological treatment as the observational study protocol does not decree specific medication or medication doses. However, this should be similar for both groups studied. In fact, the response rates in the two groups are similar, and the group differences in SNAP-IV scores seem smaller at 3 months follow-up despite higher SNAP-IV scores at baseline in the ASD group. This would mean that if anything we are underestimating the symptom reduction in the ASD group rather than the opposite.

The finding that the ASD group had higher levels of ADHD symptoms was not expected [33, 45], but may partly be explained by the group age difference, as well as the ASD group representing children with more severe functional deficits. However, adjusting for age did not change the main results for this study.

The results from the present study are in line with the results from the only previous study directly comparing ADHD medication effects in children with and without concomitant ASD [33]. Neither in a retrospective chart review, nor in a prospective observational design did they find any statistically significant differences in treatment effect (as measured by the Clinical Global Impression- Improvement scale, CGI-I) or in side-effects.

The present study was designed to evaluate both effectiveness of pharmacological treatment for ADHD as well as analysis of adverse events arising from medication. The baseline measures for adverse events enabled us to adjust for symptoms already prevailing at treatment start, causing more robust results.

Further, we examined both the absolute reduction in ADHD symptoms from baseline to 3 months follow-up and response rate (defined as 40% reduction from baseline to 3 months) to investigate if the lack of difference between the groups remained introducing a clear cutoff. We also analyzed the SNAP-IV score subdivided in the three subscales: inattention, hyperactivity/impulsivity, and ODD. The results, for both additional examinations, was consistent, with SNAP-IV at baseline being the only significant predictor of SNAP-IV at 3 months, except for when performing linear regression for the subscale inattention, where also ADHD medication significantly improved ADHD symptoms (*R*^2^ 0.24, beta − 2.44 (CI: − 4.33 to − 0.55; *P* = .0011)). We hypothesize ADHD medication would also significantly have affected our original model if the number of study objects had been larger and the rate of missing information on ADHD medication had been smaller.

Importantly, we did not find any significant difference in the gender ratio between the non-ASD and the ASD group or concerning pharmacological treatment at 3 months, which is positive since it has been indicated in previous studies that females with ADHD may be more easily missed in the diagnostic process and less likely to be prescribed medication [46].

The study is subject to several limitations. First, there is no control group and, consequently, this study did not randomize patients to treatment or no treatment, thus introducing a selection bias. As always in observational studies, residual confounding may influence the results.

Second, we did not have documentation as to whether or not the child had a clinical diagnosis of ASD according to the DSM-5; rather, we only noted the presence of ASD symptoms in accordance with a parent-reported symptom-assessment with ASSQ. As such, using the ASSQ scale for distinction of the ASD and the non-ASD group as well as for determining the cut-off value could be questioned [37, 39]. There is a risk of misclassification of patients, including patients without an ASD diagnosis in the ASD group and vice versa, which could result in over- or underestimation of pharmacological treatment effect and adverse events for each group. However, the importance of the study remains, since the ASSQ was not meant to be used diagnostically, but as a screening tool to evaluate the presence of core ASD symptoms. In fact, we suggest it was also a study strength that ASD diagnosis was not necessary for inclusion, as it led to a higher number of patients suffering from ASD symptoms earlier being identified, possibly prohibiting the risk of so-called ‘doctor’s delay’ [47, 48]. Nonetheless, since previous studies often defined ASD differently, definite comparative conclusions should be made with caution.

Third, our cohort was constituted of a limited number of patients and a heterogenous group, which may affect the possibility to detect any less pronounced differences between the groups. On the other hand, our study, with relatively few exclusion criteria, represents the clinical setting and resembles so-called real-world evidence. Thus, the study is expected to have proper face-validity.

Fourth, like many clinical trials, our study suffered from losses to follow up, especially concerning completion of the ADHD medication form. However, there were no significant difference in the rate of continued pharmacological treatment at 3 months between the ASD group and the non-ASD group. Neither was there a significant difference in the number of patients defined as having ASD symptoms (ASSQ points ≥ 17) between patients lost to follow up (27%) and included patients (21%) (*P* = .11), thereby diminishing the risk of selection bias in data. Occasional missing data points in the P-SEC scale were regarded as zero, which might have underestimated the number of adverse events. However, missing data points in that dataset were low, which implies that the risk of undervaluing the number of adverse events was small.

Fifth, we did not have any information about how many of the patients who were offered to participate in the study consented and how many declined.

Sixth, the study lacks information on pharmacological dosage, which could potentially mask a significant difference between the ASD and the non-ASD group. Hypothetically, the two groups could have been treated with different dosages. However, since pharmacological treatment is subject to clinical guidelines, it is not likely that a substantial amount of patients would have been on noticeable deviant dosages. Though, it is possible that occasional patients were treated with more than one substance. We did not aim to answer the question whether there was a difference in efficacy amidst different substances between the groups, which is indicated in other studies [15, 20, 24, 28, 49]. However, as shown in Table 1, the majority of patients, both in the Non-ASD and the ASD group, used MPH as pharmacological treatment, and no significant differences in the distribution of different substances were observed between the groups.

Seventh, when we compared the ASD and non-ASD group in experiences of adverse events, we chose to examine side-effects divided in subcategories. Adverse events per individual were not analyzed. Therefore, it is possible that one particular individual represents many adverse events. Also, the variable adverse events provided information about if clinically significant adverse events prevailed but not about the exact quantity or grading of the side-effects.

Eighth, we also have no information regarding additional treatment interventions that could interact with the results.

Ninth, mean age at inclusion was significantly 1.49 years younger in patients in the ASD group (10.26 years (CI: 9.51 to 11.04, *P* = .001)) compared to the non-ASD group (11.75 years (CI: 11.34–12.15, *P* = .001, Table 1)). We interpret this finding as logical and reasonable considering that children with more symptoms tend to be noticed earlier. Nonetheless, age did not significantly affect outcome in any of our models.

Tenth, in our study, we were limited to use only SNAP-IV Parent rating scale as a proxy for ADHD symptoms. Since children may behave different at home compared to in a school environment, having access to the SNAP-IV Teacher rating scale, could have given a more complete picture. However, additional informants also mean greater challenges to carry through data collection with the risk of less participation rate. Including teachers’ ratings in future data collections will be an asset to study any differences between the groups in the school setting.

Despite limitations, we believe that our results are an important contribution to research aimed at examining the effect of pharmacological ADHD treatment in children with comorbid ASD or ASD symptoms. For clinicians as well as for experts stipulating medical guidelines, it is crucial that decisions are based on best available evidence, increasing the probability of patients receiving the appropriate treatment. ADHD is associated with lower quality of life [50]. Comorbidity with ASD or high level of ASD symptoms does not ease this burden [51, 52]. If a misconception exists that patients with pronounced symptoms of ASD might not benefit from pharmacological ADHD treatment, they are at risk of not obtaining an efficient regimen. A previous epidemiological study found differences in prescription patterns between individuals with ADHD and individuals with ADHD and comorbid ASD [49], although what underlies these differences is not known.

Our results indicate the need to reconsider that ASD patients experience more adverse events, which could precipitate preterm pharmacological treatment, for symptoms that are not related to medication but to the characteristics of the diagnosis itself. The need of equal and correct treatment for children suffering from comorbid disorders is essential, and the possibility of customized treatment must be further elucidated. Since our study is the second to directly compare the pharmacological effect of ADHD medication between ADHD patients with different levels of ASD symptoms, more research is needed to shed light on the discrepancy of our results and previous perceptions.

## Conclusions

In summary, our results indicate that there were no significant differences in pharmacological treatment effect between patients with ADHD and concomitant pronounced ASD symptoms and patients with ADHD without high level of ASD symptoms. There was also no significant difference in the number of reported clinically significant adverse events between the groups. Studies directly comparing the effects of ADHD medication in children with only ADHD and children with comorbid ASD are important to diminish the risk that decisions about medication rely on unconfirmed assumptions. The present results are in line with the results from the only previous prospective study comparing these patient groups.

## Data Availability

Due to legal reasons and the ethical permit for the study, the data that support the findings of this study are not publicly available. According to Swedish regulations, the data are classified as sensitive personal data. The data are accessible on reasonable request from the corresponding authors. Raw data are generated at Karolinska Institutet.

## Abbreviations

ADHD: Attention-deficit/hyperactivity disorder
ASD: Autism spectrum disorder
SNAP-IV: Swanson
PDD: Pervasive Developmental Disorders; Nolan and Pelham Teacher and Parent ADHD rating scale-version IV
ASSQ: Autism Spectrum Screening Questionnaire
P-SEC: Pediatric Side Effects Checklist
ODD: Oppositional defiant disorder
DSM-V: Diagnostic and Statistical Manual of Mental Disorders
Mis: Multiple imputations
MCMC: Markov Chain Monte Carlo method
OCD: Obsessive compulsive disorder
MPH: Methylphenidate
EDX: Dexamphetamine
LDX: Lisdexamphetamine
ATX: Atomoxetine
GXR: Guanfacine
ID: Intellectual disability
